# Intra- and Interday Reliability of Spine Rasterstereography

**DOI:** 10.1155/2013/745480

**Published:** 2013-06-02

**Authors:** Laura Guidetti, Valerio Bonavolontà, Alessandro Tito, Victor M. Reis, Maria Chiara Gallotta, Carlo Baldari

**Affiliations:** ^1^Department of Movement, Human and Health Sciences, University of Rome “Foro Italico,” Piazza Lauro De Bosis 15, 00135 Rome, Italy; ^2^Department of Sports Science, Exercise and Health, Trás-os-Montes e Alto Douro University, 5001-801 Vila Real, Portugal

## Abstract

To determine intra- and interday reliability of spine rasterstereographic system Formetric 4D with and without reflective markers. Twenty-six healthy volunteers (M group) had two markers placed in correspondence of vertebra prominens and intergluteal cleft, and 24 volunteers (NM group) were assessed without markers. All participants were analyzed two times in the same day and one time on a separate day. Trunk length, kyphotic angle, lordotic angle, pelvic inclination, kyphotic and lordotic apex, right and left lateral deviation, *flèche cervicale* and *lombaire*, trunk imbalance, pelvic tilt, inflection point, rotation correction, right and left surface rotation, pelvic torsion, and trunk torsion were measured. Intraclass correlation coefficient (ICC) and Cronbach Alpha (C**α**) were calculated. In M group, for intra-, interday, and overall evaluations, the higher reliability coefficients were 0.971, 0.963, and 0.958 (ICC) and 0.987, 0.983, and 0.985 (C**α**) for trunk length, kyphotic angle, and lordotic apex, respectively; while in NM group, they were 0.978, 0.982, and 0.972 and 0.989, 0.991, and 0.991 for trunk length. In M group, the lower values were 0.598, 0.515, and 0.534 (ICC) and 0.742, 0.682, and 0.784 (C**α**) for trunk and pelvic torsion and in NM group 0.561, 0.537, and 0.461 and 0.731, 0.695, and 0.729 for left lateral deviation. The reliability of most parameters was excellent.

## 1. Introduction

Radiologic investigation is still considered the gold standard method for analyzing spine deformities, but in the last thirty years noninvasive methods have been studied for analyzing the spine, such as, for example, rasterstereography [1–3]. Rasterstereography is an investigation method developed by Drerup and Hierholzer [4] in the 1980s that allows the tridimensional reconstruction of the thoracic and lumbar spine starting from the back surface analysis. The Formetric 4D (DIERS, International GmbH, Schlangenbad, Germany) is a largely used rasterstereographic system.

There have been previous studies which evaluated the validity of the Formetric 4D, in terms of accuracy and reliability. Mohokum et al. [5] concluded that the determination of the reliability, that is, the reproducibility, for the Formetric 4D system, is essential, as previous studies had only evaluated the accuracy of the system comparing rasterstereographic evaluations to anterior-posterior radiographs [6–8]. In their study Mohokum and colleagues [5] calculated the intratester reliability for kyphotic and lordotic angles, trunk length, and trunk inclination: they concluded that rasterstereography method has a good reliability in healthy volunteers and also found that BMI does not influence the rasterstereographic reliability.

Rasterstereography is used to help long-term follow-up for the clinical patients to verify the effectiveness of surgical or conservative therapy, allowing a meaningful reduction of X-ray exposition. It is then essential that the method provides reliable information across the different days of examination. It is also to verify the reliability of rasterstereography for repeated measurements conducted in the same day to verify the effectiveness of a certain treatment.

Therefore, this study aimed to verify the intraday and interday reliability for a large number of parameters that are presented below.

The aims of this investigation were two.

1. To assess the intraday and the interday reliability, the same expert operator performed two repeated measurements two hours apart each other in the same day; one more measurement was performed on a separate day, one week apart from the previous two, the same day of the week, and at the same time of the first intraday evaluation.
2. To verify if the reflective markers could enhance or not the reliability of the rasterstereographic examination.

## 2. Materials and Methods

Fifty healthy volunteers (23 ± 3 years of age) were recruited and randomly divided into two groups: one group (M, *n* = 26) had two reflective markers manually placed in correspondence of vertebra prominens (VP) and of sacrum point (SP) at the beginning of intergluteal cleft, while the other group (NM, *n* = 24) was assessed without markers. All participants were analyzed two times in the same day (intraday evaluation) and one time on a separate day (interday evaluation). All subjects (36 males and 14 females, 23 ± 3 and 22 ± 2 years of age, resp.) were evaluated with DIERS Formetric 4D using DiCam II Software in 4D Average modality. This modality allows an average measurement based on 12 subsequent images in a time interval of six seconds.

Subjects were asked to not perform any physical or sport activity before the evaluations, nor in between the two intraday tests.

To standardize subjects' positioning, a metallic bar was placed on the floor in order to provide a reference for the subjects' feet. Subjects were positioned in a standing, upright position, with their arms naturally left along the hips and were prepared to the analysis as follows:

- the patient was undressed to his/her (under) pants for the measuring procedure. During the measurement the entire buttocks are revealed; that is, the (under) pants are positioned right under the bottom;
- in case of long hair, they were tied up with a suitable means (cap, hair clips, hair bands, etc.) so that the neck is visible up to the hairline;
- rings, watches, and necklaces in particular were removed to prevent the occurrence of any reflections from the lines of light on the one hand and artificial changes (necklace increases the likelihood) on the other hand.

In order to improve the accuracy of the machine, as suggested by the manufacturer for the dynamic and average analysis modalities, we manually put two reflective markers on the subject's back: the first one was placed in correspondence of the “vertebra prominens” (VP), and the second one in correspondence of the sacrum point (SP) at the beginning of the intergluteal cleft. The local university ethic committee approved this investigation.

The following parameters were measured and taken into consideration for the present study: trunk length VP-DM (detected from vertebra prominens (VP) to midpoint of lumbar dimples (DM)), kyphotic angle ICT-ITL max (measured between tangents of cervicothoracic junction (ICT) and of thoracolumbar junction (ITL)), lordotic angle ITL-ILS max (measured between tangents of thoracolumbar junction (ITL) and of lumbosacral junction (ILS)), pelvic inclination, kyphotic Apex, lordotic Apex, lateral deviation VP-DM (right), lateral deviation VP-DM (left), *flèche cervicale*, *flèche lombaire*, trunk imbalance (°), trunk imbalance (mm), inflection point ITL, inflection point ILS, surface rotation (right), surface rotation (left), pelvic torsion DL-DR (°) (calculated from the torsion of the surface normal on left (DL) and right (DR) lumbar dimples), and trunk torsion.

### 2.1. Statistical Analysis

Intraclass correlation coefficient (ICC) and Cronbach Alpha (C*α*) were calculated using PASW Statistics version 18 software package for Windows (SPSS Incorporation, Chicago, IL).

The ICC was selected because, as suggested by Bartko [9] and Shrout and Fleiss [10], “it provides the most conservative estimate of reliability (or reproducibility) because it is high only when the variance among trials for a particular subject is small, relative to the variance within a trial.” ICC may range from −1 to +1 [11]. According to Rosner [12], ICCs less than: ±0.40, indicate poor reliability; ±0.40–0.75, fair or good reliability; and ±0.75–1.00, excellent reliability. Referring to C*α*, George and Mallery [13] provide the following rules of thumb: “>.9—Excellent, >.8—Good, >.7—Acceptable, >.6—Questionable, >.5—Poor, and <.5—Unacceptable.”

It should also be noted that an Alpha of .8 is probably a reasonable goal. All parameters were analyzed simultaneously using a multivariate analysis of variance (MANOVA) to verify the effect of both markers positioning and of the typology of parameter (invariant, variant) on the intraday, interday, and overall reliability.

## 3. Results

In Tables 1 and 2, the results for ICC and C*α* values for each measured parameter for M and NM groups, respectively, for intraday and interday evaluations are shown.

In M group ICC ranged between 0.971 (trunk length) and 0.598 (trunk torsion) for intraday evaluations, between 0.963 (kyphotic angle) and 0.515 (pelvic torsion) for interday evaluations, and between 0.958 (lordotic apex) and 0.534 (pelvic torsion) overall.

Instead, for C*α*, values ranged between 0.987 (trunk length) and 0.742 (trunk torsion) for intraday, between 0.983 (kyphotic angle) and 0.682 (pelvic torsion) for interday, and 0.985 (lordotic apex) and 0.784 (pelvic torsion) overall.

In NM group ICC ranged between 0.978 (trunk length) and 0.561 (left lateral deviation) for intraday evaluations, between 0.982 (trunk length) and 0.537 (left lateral deviation) for interday evaluations, and between 0.972 (trunk length) and 0.461 (left lateral deviation) overall.

Instead, for C*α*, values ranged between 0.989 (trunk length) and 0.731 (left lateral deviation) for intraday, between 0.991 (trunk length) and 0.695 (left lateral deviation) for interday, and between 0.991 (trunk length) and 0.729 (left lateral deviation) overall.

## 4. Discussion

The aim of this study was to evaluate the intra- and interday reliability of Formetric 4D and secondly to verify the influence of the reflective markers on the reliability.

ICC and C*α* were found to be paired for the same parameter for both the highest and lowest values: highest values for both ICC and C*α* were found for trunk length, kyphotic angle, and lordotic apex: these parameters are defined invariant, that is, not dependent on the subject's positioning with respect to the machine; this is in line with an investigation by Hackenberg et al. [14] who suggested to use mainly the Average 4D modality, in order to reduce the measurement error deriving from the patient's positioning; lowest values were found for trunk torsion for the intraday evaluations and for pelvic torsion for both interday and overall. This result is not in line with an investigation by Goh et al. [15], where ICC values ranged between 0.98 and 0.99. This could be related to two aspects: first of all, pelvic and trunk torsions are both variant parameters, dependent on patient's positioning; therefore, despite the indications given to the subjects analyzed, a greater range of error is reasonable; second of all, these are parameters not directly measured, but calculated as measures deriving from the invariant parameters; this double calculation may lead to a greater measurement error. Moreover, lowest values were obtained for parameters related to torsion, which is very dependent on the patient initial position, even if subjects were all instructed on this issue for every evaluation performed.

In NM group, anyway, according to George and Mallery [13], lowest mean value for C*α* was considered good (0.898) and lowest single parameters value was anyway acceptable (0.695) for interday evaluation of left lateral deviation; in M group mean value for C*α* was almost excellent (0.883) and lowest value for interday evaluation of pelvic torsion was fairly acceptable (0.682). According to Fleiss' guidelines [16], ICC lowest mean value in NM group was excellent (0.824) and lowest value for single parameters was good for the interday evaluation of left lateral deviation (0.537); instead in M group lowest mean value was excellent (0.797) and lowest single parameter value was good for interday evaluation of pelvic torsion (0.515).

In M group, for intraday, all the parameters had an ICC considered excellent, except for right lateral deviation, and right surface rotation pelvic and trunk torsions (anyway considered almost good or fair); for interday all parameters had an excellent ICC, with the exception of left and right lateral deviation, left and right rotate surface, and pelvic torsion, that ranged between 0.40 and 0.75, anyway considered almost good.

Referring to the highest values, for intraday, all the parameters had an ICC considered excellent, except for inflection point ITL, pelvic rotation, and pelvic torsion (anyway considered almost good or fair); for interday, also pelvic inclination both measured in degree and in mm, left lateral deviation, and trunk torsion had an ICC that ranged between 0.40 and 0.75, possibly because of the necessity of repositioning the two markers between the two days of evaluation. Our findings are in line with Mohokum et al. [5], who found excellent values of reliability for the intraday evaluations without any marker positioning for those invariant parameters taken into consideration in their study (trunk length, trunk inclination, and kyphotic and lordotic angle); in our study, a larger set of invariant parameters were studied and showed an excellent reliability, but also variant parameters were taken into consideration, showing a good reliability. Moreover, MANOVA results showed that the presence of the markers did not significantly influence neither the intra- nor the interday evaluations. The present study suggests that the presence of the markers is not necessary for the intraday evaluations but can play a disturbing role for the interday evaluations, because of the repositioning process. Therefore the use of markers is advisable only for the dynamic modalities of evaluation, as it is suggested by Formetric's manufacturer. Type of parameter (variant versus invariant), instead, systematically affected the measure of reliability; anyway, even if invariant parameters showed greater reliability, variant parameters had reliability values considered almost good.

Therefore, an acceptable reliability also for the lowest values, both for ICC and for C*α*, was found.

## 5. Conclusions

In conclusion, the present study reveals a good to great reliability of the DIERS Formetric 4D system depending on the typology of the measured parameter. Therefore, this study validated aspects of the rasterstereographic measuring system that potentially could replace X-rays in follow-up of spinal deformities helping to reduce X-rays irradiation.

## Figures and Tables

**Table 1:** ICC and C*α* values for intraday, interday, and overall repeated measures of M group.

Parameter	Intraday		Interday		Overall	
	C*α*	ICC	C*α*	ICC	C*α*	ICC
Trunk length*	0.987**	0.971**	0.963	0.931	0.981	0.943
Kyphotic angle*	0.976	0.951	0.983**	0.963**	0.984	0.954
Lordotic angle*	0.957	0.912	0.962	0.928	0.972	0.919
Kyphotic apex*	0.917	0.845	0.943	0.896	0.961	0.893
Lordotic apex*	0.983	0.965	0.975	0.952	0.985**	0.958**
Flèche cervicale*	0.958	0.920	0.950	0.907	0.956	0.881
Flèche lombaire*	0.934	0.869	0.868	0.774	0.921	0.796
Trunk imbalance* (°)	0.961	0.923	0.924	0.861	0.956	0.879
Trunk imbalance* (mm)	0.868	0.760	0.855	0.748	0.901	0.737
Pelvic inclination* (mm)	0.954	0.914	0.828	0.700	0.896	0.736
Inflection point ILS*	0.985	0.970	0.970	0.944	0.984	0.952
Inflection point ITL*	0.842	0.734	0.841	0.725	0.871	0.695
Left Lateral deviation	0.887	0.772	0.739	0.596	0.838	0.643
Right Lateral deviation	0.903	0.829	0.932	0.877	0.944	0.852
Left Surface rotation	0.905	0.832	0.922	0.851	0.944	0.849
Right Surface rotation	0.884	0.797	0.869	0.762	0.911	0.766
Pelvic torsion	0.865	0.748	0.682***	0.515***	0.784***	0.534***
Trunk torsion	0.742***	0.598***	0.811	0.625	0.816	0.568

Mean	0.915	0.849	0.883	0.797	0.917	0.797

*Invariant parameters.

**Highest value.

***Lowest value.

**Table 2:** ICC and C*α* values for intraday, interday, and overall repeated measures of NM group.

Parameter	Intraday		Interday		Overall	
	C*α*	ICC	C*α*	ICC	C*α*	ICC
Trunk length*	0.989**	0.978**	0.991**	0.982**	0.991**	0.972**
Kyphotic angle*	0.965	0.927	0.906	0.768	0.923	0.802
Lordotic angle*	0.956	0.918	0.971	0.945	0.975	0.930
Kyphotic apex*	0.955	0.917	0.972	0.947	0.971	0.919
Lordotic apex*	0.982	0.962	0.982	0.966	0.982	0.948
Flèche cervicale*	0.979	0.960	0.945	0.900	0.972	0.922
Flèche lombaire*	0.955	0.903	0.962	0.929	0.968	0.906
Trunk imbalance* (°)	0.920	0.857	0.950	0.907	0.951	0.870
Trunk imbalance* (mm)	0.969	0.943	0.959	0.923	0.974	0.928
Pelvic inclination* (mm)	0.940	0.892	0.950	0.905	0.961	0.894
Inflection point ILS*	0.966	0.935	0.986	0.972	0.977	0.935
Inflection point ITL*	0.857	0.758	0.861	0.761	0.870	0.696
Left lateral deviation	0.731***	0.561***	0.695***	0.537***	0.729***	0.461***
Right lateral deviation	0.774	0.641	0.805	0.672	0.777	0.542
Left surface rotation	0.883	0.795	0.832	0.721	0.885	0.726
Right surface rotation	0.808	0.686	0.782	0.630	0.806	0.580
Pelvic torsion	0.823	0.704	0.741	0.598	0.828	0.624
Trunk torsion	0.752	0.607	0.890	0.777	0.833	0.622

Mean	0.900	0.830	0.898	0.824	0.909	0.793

*Invariant parameters.

**Highest value.

***Lowest value.
